# Ancient Food Habits Dictate that Food Can Be Medicine but Medicine Cannot Be “Food”!! *—“Food is the substance digested by an individual* *to suppress the basic vital instinct of hunger”* *(H.K.G)*

**DOI:** 10.3390/medicines4040082

**Published:** 2017-11-13

**Authors:** Hit Kishore Goswami, Hitendra Kumar Ram

**Affiliations:** 1 Retired Professor of Botany & Genetics, 24, Kaushalnagar, P.O. Misrod, Bhopal (MP) 462026, India; 2 Department of Biodiversity Conservation and management, ABVH University, Bhopal (MP) 462001, India; ramhitendra@gmail.com

**Keywords:** edible fruits in the wild, edible ferns, plants as ancient food, plants offer medicinal compounds, medicines are not food, phytochemicals, pharmaceutical industry, edible fruits, roots and leaves as food-therapy

## Abstract

**Background:** Extensive surveys of several population settlements in different parts of India—covering plains, mountains, valleys, river banks and deeper areas of forests at different altitudes—between 1968 and 2016 demonstrated that the basic vital need of hunger is being fulfilled since antiquity by plants in the wild. **Methods:** Based on collections, consultations with local population personnel and literature searches, this paper presents many plants that are commonly used as food and focuses on their products, which are rich in alkaloids, polysaccharides, steroids, terpenoids, flavonoids, aminoacids, fatty acids and antibiotics etc. These complex organic compounds are suitable for the production of drugs for many ailments/diseases, including the prevention of cancers. **Results:** There are more than 100 families including several hundred plant taxa from various plant groups like angiosperms, bryophytes, pteridophytes, gymnosperms and even fleshy fungi, which have offered essential food items to ever-growing human populations since antiquity. Phytochemicals functioning as antioxidants are exceedingly beneficial to the human body but excess consumption of these compounds, adding higher levels of antioxidants, may even be responsible for chronic diseases including aging, cancer, cardiovascular diseases, rheumatoid arthritis, atherosclerosis, etc. These medicines can obviously be taken in small and prescribed quantities but can never be consumed as “food items.”

## 1. Introduction

Ingestion of food is an inherently basic instinct of all life forms; each one, small or big, needs water and food to survive. The entire biological web has survived and is recycled within the food chain. Instinct of “eating” or “being fed” is inborn; even a few hours old child grabs his palm and takes it to the mouth. A normal, growing child continues to grab just anything and takes it to the mouth thereby proving universality of intake of food for survival. Here, we can query, what is food? Primarily, “Food can be defined as the material or substance which can be eaten, digested and regularized for feeling satiated without any visible immediate harm.”The majority of humans migrated outside their shelters to open areas and settled as communities and gradually installed the concept of societies but there have always been lots of settlers in and around forests, which today reveal their presence. Temporary shelters and moving populations are observed in several parts of the world. Isolated populations prevalent in many parts are known as “tribes” in India. Early human populations were migrating for survival across forests in search of the fundamental requirements of life: water, food and shelter. After several centuries of migration, certain tribes settled by the side of rivers or water resources and searched for shelter inside caves or rock shelters. These population fractions must have been descendants of enlightened and intelligent individuals who were able to discover the specific identification of good and bad plants, healthy, tasteful and poisonous plant parts and small animals. Hunting habits and selective food habits along with safety survivals were the lessons those migrating populations must have conveyed or passed on to their young and accompanying companions. This phase of human progress, where forest produce became an indelible centered-dogma for human survival, must have taken several thousand years to become a part of human society. Food intake must have depended on the availability of plants and animals around these populations. So, the evolution of food habits was necessitated by environmental surroundings. Even today, this is the most validated concept. Since that time, thousands of plant species of all forms—trees, shrubs, herbs, lianes (lianas) and aquatic plants—that are prevalent in all kinds of ecosystems, have been meticulously exploited for food, fodder and medicines [1,2,3,4,5,6,7,8,9,10,11,12]. The leaves/stem/roots/corms/flowers or fruits of traditional food plants have been uninterruptedly used as supplements to cereals and rice and are known to be prepared in various forms such as decoction, curry, mixed with vegetables, used as tea or similar drinks and also as additional food items with bread and baked food stuffs. This paper presents many plants commonly used as food and focuses on plant products that are often rich in classical compounds (antioxidants, proteins, carbohydrates, minerals, etc.) now being extracted and used as life-saving drugs.

**Figure 1:**
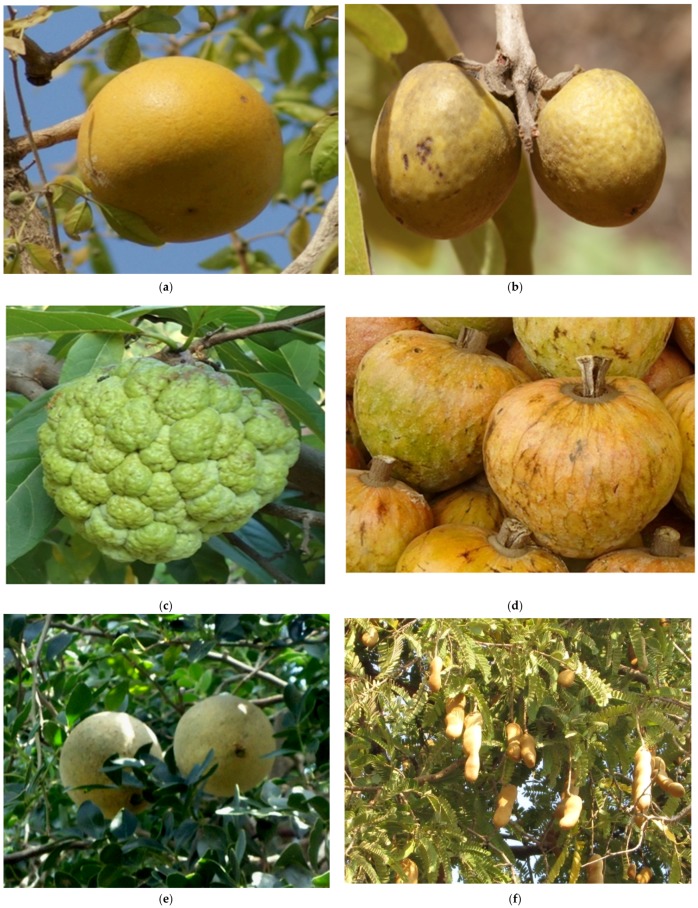
Edible Fruits in Natural Forests: (**a**). *Aegle marmelos* (L.) Corr; (**b**) *Diospyros melanoxylon* Roxb; (**c**) *Annona squamosa* Linn; (**d**). *Annona reticulata* Linn; (**e**) *Feronia limonia* (L.) Swingle; (**f**) *Tamarindus indica* Linn.

**Figure 2:**
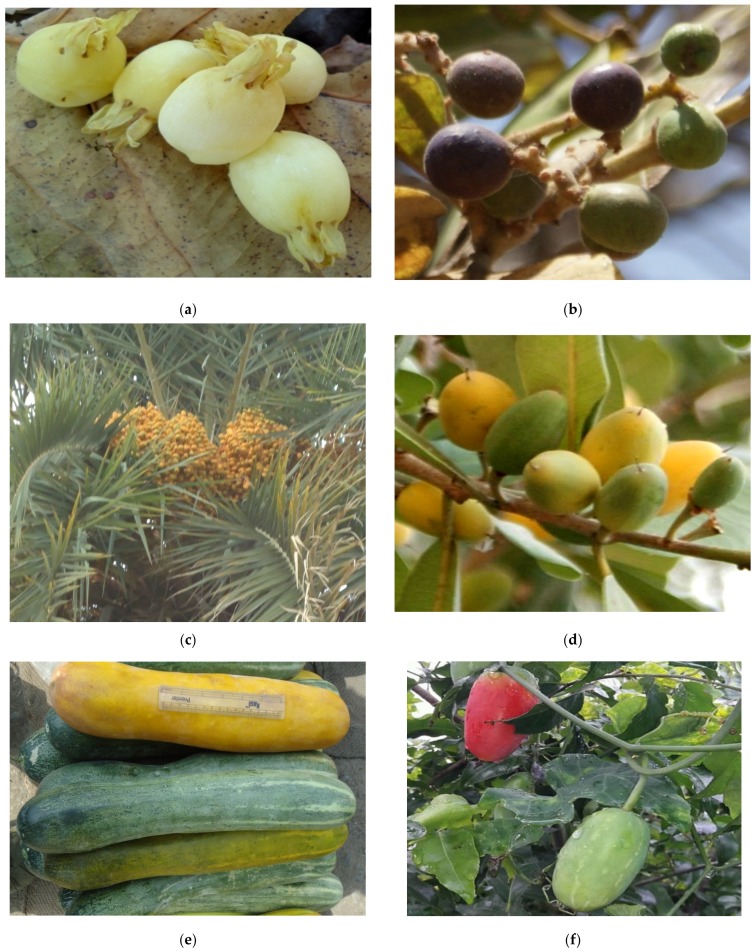
Edible Fruits in the wild/forest areas and villages: used as supplement to food (**a**) *Madhuca latifolia* J.F. Gmelin; (**b**) *Buchnania lanzan* Spreng; (**c**) *Phoenix sylvestris* Roxb; (**d**) *Manilkara hexandra* (Roxb.) Dubard; (**e**, *Cucumis sativus* ; **f**, *Coccinia grandis*).

**Figure 3:**
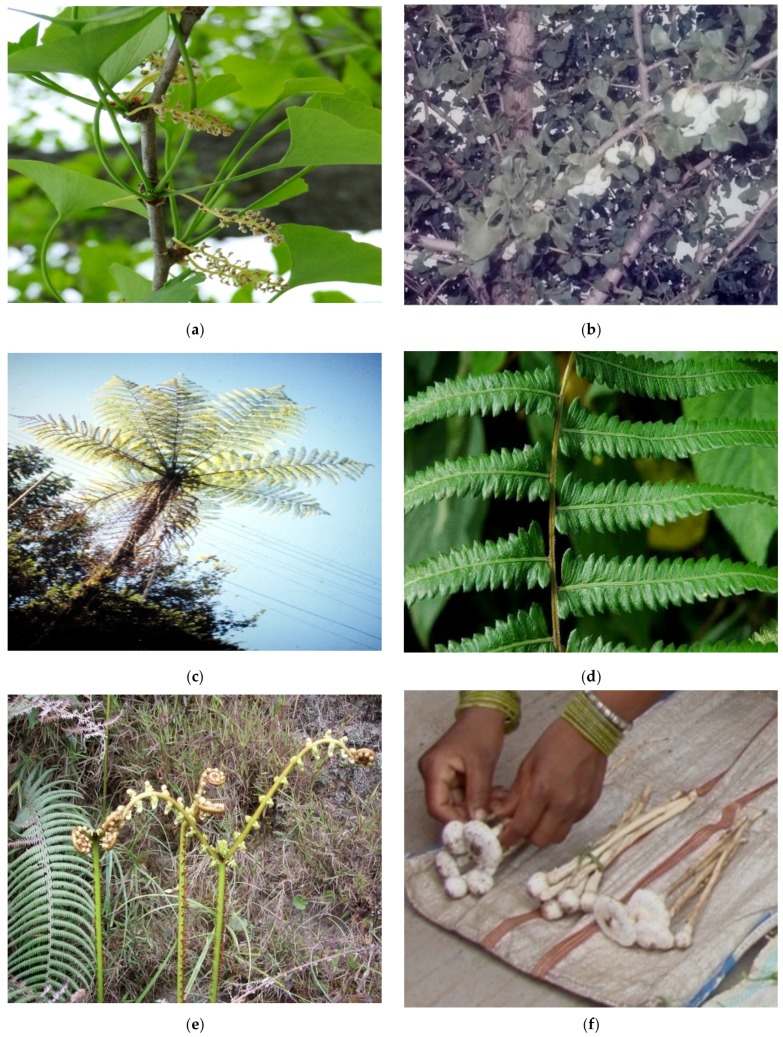
Plants in Nature producing Edible leaves and reproductive parts: (**a**,**b**) *Ginkgo biloba* male and female plants; ovules are hanging on a twig of (**b**,**c**) Tree fern *Cyathea* a common tree fern offers small soft young twigs; (**d**) Fronds of fern, *Ampelopteris prolifera*; (**e**) Note bunch of small soft young circinately coiled leaves and stem parts in *Gleichenia glauca*; (**f**) Edible mushroom, *Termitomyces eurrhizus*. Syn. *Termitomyces albuminos.*

**Figure 4:**
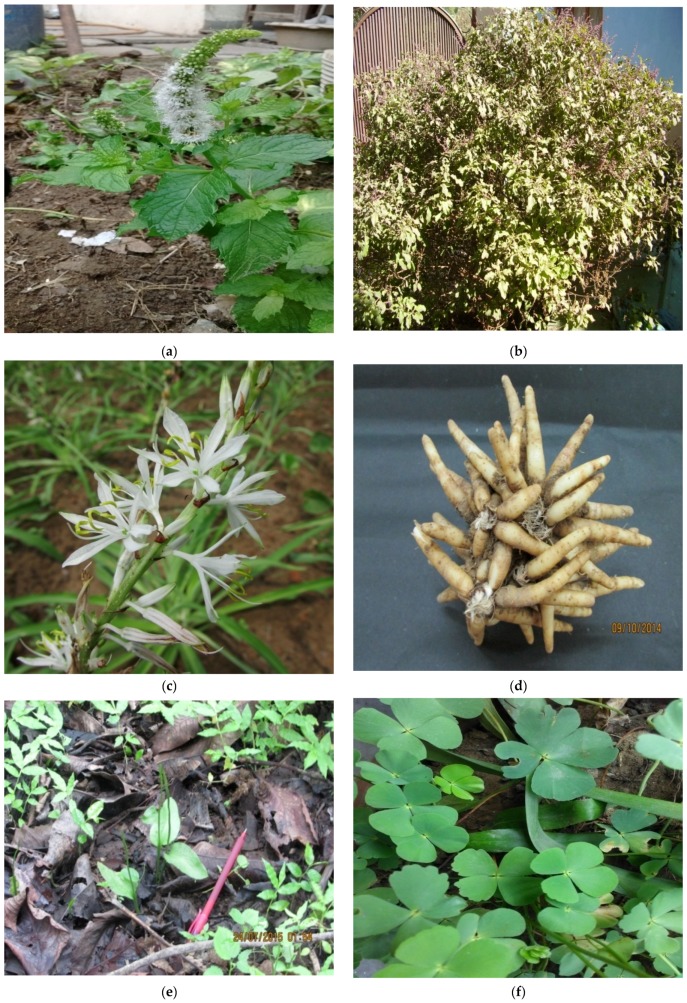
Herbaceous edible plants (mostly leaves): (**a**) *Mentha spicata*; (**b**) *Ocimum sanctum*; (**c**) *Chlorophytum tuberosum* with flower; (**d**) Roots of *Chlorophytum tuberosum*; (**e**) *Ophioglossum reticulatum;* (**f**) *Marsilea minuta.*

Within the past few decades we have discovered that these plants yield an appreciable quality and quantity of alkaloids, polysaccharides, steroids, terpenoids, flavonoids, aminoacids, fatty acids and antibiotics, which are suitable for the production of drugs for many ailments/diseases including the prevention of cancers [12,13,14,15,16,17,18,19,20,21]. Precisely, our list includes those edible plants routinely used as food and that these are rich in active antioxidant systems for self-protection against biotic and abiotic stress conditions. These antioxidant activities of plants have useful effects on human bodies. As is well established, the most important function of antioxidants is reducing reactive oxygen species (ROS). ROS are a byproduct of respiration. They can be beneficial to the human body by removing pathogens and old proteins. However, an overproduction of ROS (which can be due to various factors such as environmental pollutions; extra dose of antioxidants etc.) may cause stresses and related problems. These situations could eventually be responsible for chronic diseases including aging, cancer, cardiovascular diseases, rheumatoid arthritis, atherosclerosis, etc. Needless to say, the list of chemical compounds extracted by modern pharmaceutical research from these food-plants (some are listed in Table 1 and Table 2, Figure 1, Figure 2, Figure 3 and Figure 4) demonstrate that ancient food habits were health oriented. These drugs or new generations of chemical compounds in the form of medicines, extracted from food items, cannot be consumed as food in bulk every day. Certainly, any “medicine/tonic” cannot be eaten to satisfy “hunger” or fulfill all physiological needs necessary for the growth and regular maintenance of bodily functions.

### 1.1. Types of Food: Definition and Classification of Foods

Ethnic communities have been almost always engaged in grim battle for survival. The life of these ethnic communities is closely woven around the forest from pre-historic days. These communities depend entirely on plant resources, which grow wildly and abundantly in the environment. Due to their semi-nomadic life and the shifting of natural resources, traditional agricultural practices are not common. They are solely dependent on forest for their survival. Plant products like roots, leaves, fruits etc. constitute their food. Like other tribal groups found in various parts of India, the interaction of these ethnic people/tribes with the surrounding vegetation is greatly evident as they extensively use them for the construction of their houses, as fodder for their cattle and also many plants are used to prepare local beverages. Interestingly, some of the plants are considered as sacred and their religious rituals revolve around them, even the names of the many trees are used as symbols by various tribal communities. The most interesting aspect of the life of ethnic people is the utilization of plant products in their health care systems. It is intriguing that many botanically lower group-plants (such as mosses, lichens and ferns) and many angiosperms (flowering plants) have been mentioned as medicines for centuries in ancient literatures in different countries (China, Korea, India) [6,9]. This fact has been known to tribal communities found in various parts of India and elsewhere [5,10,32,33,34,35,36,37]. In fact, there have been hundreds of publications along these lines, which obviously indicate that knowledge regarding the importance of many such plants as medicines [see reviews: [18,37,38] has been socially inherited for hundreds of generations.

In India, aborigines (tribal folk) inhabiting the Andaman and Nicobar Islands are among the most primitive people and ethnomedicinal information from these people has engendered a lot of scientific curiosity and very surprisingly these plant genera included many pteridophytes (ferns and related group of plants). As is well established, the tribal groups inhabiting the Andaman & Nicobar Islands are the oldest human population, which settled around 35 to 60thousand years ago, [1,5,10,37,38] who have been known to consume the leaves and roots of certain plants, which have proven to be highly effective as medicines. The eating of leaves and roots might have been supplementary food items but their regular use has been established as a social system. According to Pandey et al. [10] more than 2000 plant species are known to these tribal groups—there are hundreds of species whose leaves, fruits and/or roots are selectively used orally before or after dietary intake or as per need of the body. This is a hard fact that they have been well versed with the medicinal uses of these plants.

Obviously, it becomes a plausible inference that early populations had tried to satisfy their hunger by randomly eating plant parts and many such attempts must have caused harm because of the toxic nature of some plants. By experience, such plants were labeled as toxic and such knowledge was intelligently transferred to younger folk. Indian Aayurvedic literature for centuries has been aware of such detailed classifications of edible and non-edible plants. A recent study [39] points out, through their survey of an ancient herbarium of Francesco Bolòs—who had presented plants used in 18th and 19th century as revealed in historical ethnobotany—a total of 385 plant specimens (381 taxa) have been detected bearing medicinal use and information on folk names. Their publication reports onthe medicinal properties of plants (in Latin), which includes 32 indications of toxicity, nine reports of food use, and 123, 302 and 318 popular plant names in Catalan, Spanish and French, respectively. The most quoted systems are digestive, skin and subcutaneous tissue (plus traumatic troubles) and genitourinary. So, the identification or labeling of certain plants as toxic and also as medicinal was on account of their being used as food and often people would have found them toxic and/or preventive.

### 1.2. Types of Food

Ethnobiologically, “Food” can be classified as:

1. Main food, which satisfies hunger; 2. Supplementary food, the type or form of food thatcan be used as additional or subsidiary to the main food (such as fruits, salad and healthy drinks—milk, juice etc.); 3. Food items—this category should include: (i) Precautionary food; (ii) Food ingredients.

## 2. Methods: Field Work and Description

### 2.1. Main Food

Extensive field work in various kinds of forests at different altitudes and in different seasons over more than five decades has [6,37,40,41,42,43] shaped genuinely relevant experience that the essential survival of biota on this planet has been practically possible only due to highly diversified plant populations, which have aided the survival of not only humans but also the entire animal kingdom. Certain examples of those commonly known plants that have had played a prime role in offering foods are presented below, as well as in Table 1 and Table 2 and Figure 1, Figure 2, Figure 3 and Figure 4.

### 2.2. Trees

We briefly mention a few trees that be are very useful and medicinally nutritive (Table 1 and Figure 1 and Figure 2) fruits consumed as part of the main food for centuries. These days their usage has taken on different forms (used for pulp/juice/production etc.) but they are in universal use.

#### 2.2.1. *Aegle marmelos*(L.) Corr

This tree is found in the forests of Myanmar, Indo-china, Sub-Himalayan tract, Pakistan and all over India. Its fruits (Figure 1a) have been used in traditional medicine and also as a part of main food for centuries. The fruits can be properly stored for a year or more. During our field work in tribal areas people had reported that one ripe fruit can fulfill the average hunger of an adult. Fruit pulp (Table 1) contains sugars and digestive enzymes—a ripe fruit can be stored for use after several months as the fruit cover is hard and stony. Its fruits are astringent, digestive, possess antibiotic and laxative properties; its leaves are also antibiotic, febrifuge, and recommended for the treatment of diabetes [20]. This moderate size of tree is also cultivated currently.

#### 2.2.2. *Diospyros melanoxylon* Roxb

A very useful tree found in tropical and subtropical forests and quite common in Sri Lanka and India. Its fruits are eaten frequently (Figure 1b) for the treatment of general fatigue. Nowadays, leaves, bark and flowers are picked up by local herbalists for preparing decoction to treat urinary infections in rural practices. Its young soft leaves yield many diuretic and laxative compounds.

#### 2.2.3. *Cocos nucifera* Linn

Found throughout the Indo-Pacific region, this plant is cultivated throughout the tropics. Popular coconut trees are distributed in many forests and are now cultivated worldwide but Asian coconuts are of great nutritional value. The green coconut inner sap/fluid is rich in glucose, antiseptic constituents and generates much energy. It can be said that the coconut plant itself is a mini-industry, combining both medicinal as well as commercial potential. Not a single part of the coconut tree is without any use.

#### 2.2.4. *Madhuca indica* J F Gmel

Mainly found in Myanmar and India, this common tree is also grown in abundance in tropical and subtropical forests. Nowadays the tree is also excessively grown on forest road sides in many parts of India. Both its flowers and fruits (Figure 2a) are highly useful and are rich in fat and carbohydrates. Flowers are eaten fresh and also dried and stored as main food in parts of India. Its flowers are very rich in sugar, contain aminoacids serving as an energy tonic; also used to produce vinegar and liquors.

#### 2.2.5. *Syzygium cumini* (Linn) Skeels

Distributed in the forests of Sri Lanka, Malaysia and Australia, the genus has many wild species bearing edible fruits. Indian emigrants brought it overseas from India and it is common in tropical former British colonies. It has been introduced to the Cook Islands, Fiji, French Polynesia, Guam, Hawai’i, Florida, New Caledonia, Niue, Palau, Tonga, China, Indonesia, Malaysia, Christmas Island, Australia, Africa, India, Caribbean, and South America. *S. cumini* in particular bears millions of fruits of excellent quality—its ripe fruits are good for health and rich in alkaloids and its seeds yield one of the best possible remedies for controlling sugar levels. Its fruits have been used for centuries as a medicinal food for patients of diabetes and hypertension. Vinegar, as well as liquors, are also prepared from decoction of the pulp of the fruit. Trees are often grown in preserved forests and gardens.

#### 2.2.6. *Phoenix dactylifera* Linn

Native to the Middle East and North Africa, this species is widely cultivated and is naturalized in many tropical and subtropical regions worldwide. Commonly known as date palm (Figure 2c), these trees are the most familiar trees of arid, semi-arid and desert areas and different species are found almost everywhere. Better designated as “Berries in the Desert” or even “Diamonds of the Desert,” date palm trees are liked by inhabitants of rural and village areas because their ripe fruits are rich in sugars. Eaten both fresh and dried, the fruits are considered to be one of the cheapest widely available dry-fruits. Many fruit preparations are made such as, jams, laxatives, and expectorant; it is also used as an essential food component of milk for patients of respiratory disorders and high fevers.

#### 2.2.7. *Ficus glomerata* Roxb

This tree species is found in Bangladesh, Sri Lanka, China and Australia. Theoretically, it is originally native to Malaysia, Southeast Asia and the Indian subcontinent. The genus *Ficus* has many species bearing edible fruits (e.g., *F. heterophylla, F. hirta, F. hispida, F. lanceolata*, etc.) but among them, *F. glomerata* fruits are most routinely used as afood item in many parts of the world. Though the ripe fruits are eaten the majority of consumers prefer them roasted as they taste sweeter and act as an excellent carminative, curing stomachic disturbance. The fruits are very rich in phenolic contents, gallic acid, chlorogenic and ellagic acid. Consumption of these fruits is encouraged as they potentially have antioxidant activity, preventing DNA damage [44].

#### 2.2.8. *Ginkgo biloba* Linn

The genus *Ginkgo* is a dioecious gymnosperm (Figure 4a,b) now confined to the natural forests of China, Korea, Japan and the Malaysian peninsula. The female tree bears ovules, which are widely eaten as supplementary to dietary requirements on account of their high energy content. For more than a hundred years *Ginkgo* plants have been cultivated the world over on account of their small beautiful fan like leaves giving a graceful look to the tree and the female plants, which produce ovules in bulk. *Ginkgo* ovules are often the part of the main evening food in South Korea, Japan and China.

### 2.3. Shrubs and Small Herbs: Edible Ferns

*Ampelopteris prolifera* (Retz.) Copel. (Thelypteridaceae) *Diplazium esculentum* (Retz.) Sw. (Athyriaceae) *Marsilea minuta* L. Marsileaceae); *Gleichenia glauca,* (Gleicheniaceae) *Adiantum philippense, Acrostichum aureum* L (Adiantaceae), *Blechnum orientale* L, *Stenochlaena palustris* (Burm) Bedd ( Blechnaceae); *Ceratopteris thalictroides* Brongn, *Angiopteris evecta* (Forst) Hoffm, *Acrostichum aureum* L (Pteridaceae) and a large number of several other fern genera, including tree fern *Cyathea* spp, were eaten by early settlers in forests and even now are regular food items among tribes [5,20,37,45,46,47] in many parts of India, Malaysia and Brazil as well as in African countries. Most ferns are a rich source of phytochemicals and have been found to possess a variety of biological activities including antioxidant potential. Natural antioxidants are in high demand for application as nutraceuticals, biopharmaceuticals and food additives. This has been established by various population surveys that increased dietary intake of natural phenolic antioxidants is related to adecline in cardiovascular problems. In the tribal populations of India and elsewhere, edible ferns [32,36,48,49] have been the most preferred staple food. The high bioactivities of traditional medicinal ferns have been internationally studied and their roles have been recognized. These attempts have confirmed various bioactivities, such as an antioxidant, antimicrobial, antiviral, anti-inflammatory, anti-tumor and anti-HIV etc. The occurrence of antibiotic activity in the extracts of more than two hundred species of pteridophytes has proven to be of prime significance within the past four decades [3,13,18,37,49,50], many of them have been used as additional food items (supplementary food). Some of the fern genera possess a few unique secondary metabolites that have not been discovered in higher plants. Polyphenols are useful phytochemicals that provide health benefits such as antioxidants. Antioxidants are generally recognized to reduce the risk factors of chronic disease. In experiments screening for the total polyphenol content of several ferns, which included a few edible ferns along with *Dicranopteris pedata, Athyrium niponicum* and *Dryopteris nipponensis,* more than 13% of total polyphenols were obtained from the dried materials of both fronds and rhizomes. Rhizomes of many ferns are rich in starch and oil contents, therefore most of the early village colonizing populations had identified and used them as an equally important constituent of their main diet.

Many species of *Pteris* possess flavonoids, which are α- or β-glycosides such as flavonoid glucosides, galactosides, rhamnosides, or arabinosides. Also, Pterosins and ent-kaurane diterpenoids are the characteristic constituents of the fern family Pteridaceae [18,50] *Ampelopteris prolifera* (Retz.) Copel. (Thelypteridaceae) (Figure 3d) contain many flavonoids, including novel ones. In fact, many other edible thelypterids containaltogether new kinds of flavonoids [18,50]. *Gleichenia glauca* branches with small twigs showing circinately coiled vernation (Figure 3e) are boiled and eaten as a staple food even these days by inhabitants of some hilly areas. Phytochemicals in the family Gleicheniaceae are terpenoids, many of which exhibit distinct pharmacologically relevant activities. Flavonol glycosides are frequently present in most species of the family Gleicheniaceae. The flavonols in *Gleichenia* leaves were found to be present as 3-glucosides, 3-rhamnosides, 3-rutinosides, 3,4’-diglucosides, 7-glucosides and 7-arabinoside. Quercetin-3-glucoside was identified as a major flavonoid component in several species [51].

### 2.4. Other Herbs/Small Plants

A large number of small plants and herbs (Table 2), including ferns as mentioned above, are essential carpet flora of each and every forest ecosystem. It has been well-documented that ancient civilizations had used them for food, tea, and other drinks. Even now, there are a large number of ferns and many shrubs and herbs used as raw food (Figure 3 and Figure 4) or just cooked for eating as boiled foodstuff. The commonest example in many forest states has been observed to be the *Ophioglossum* (Figure 4e) species on the ground and *Marsilea* spp (Figure 4f) from the aquatic ecosystem. Flavonoids were isolated from the aerial parts of the pteridophyte *O. vulgatum*., including 3-*O*-methylquercetin and its glucosides, 5’-isoprenyl-3-*O*-ethylquercetin 4’,7-di-β-d-glucopyranoside, 3-*O*-methylquercetin 4’-β-d-glucopyranoside 7-[*O*-β-d-glucopyranosyl-(1 → 2)-β-d-glucopyranoside [18,52]. Seven new homoflavonoid glucosides, pedunculosumosides A–G, were isolated from the ethanolic extracts of whole *O*. pedunculosum plants. Six homoflavonoids, ophioglonin, ophioglonin 7-*O*-β-d-glucopyranoside, ophioglonol, ophioglonol prenyl ether, ophioglonol 4’-*O*-β-d-glucopyranoside, and isoophioglonin 7-*O*-β-d-glucopyranoside, quercetin, luteolin, kaempferol and 3,5,7,3’,4’-pentahydroxy-8-prenylflavone etc. [18,52].

Similarly, *Centella asiatica* on marshy land serves as a good leafy vegetable.

## 3. Supplement Food

### 3.1. Geum Quellyon Linn

Herbaceous plants were a convenient choice while searching for food. There have been a large number of such herbs which have been regularly consumed as food or supplemental food. One such example is from Chile—the then isolated populations, and even modern populations, used a herb known as *Geum quellyon* Sweet belonging to family Rosaceae because its roots contain highly aromatic compounds with a pungent odor. The roots have been used in the traditional medicine of the Mapuche Amerindians of Chile to treat gastric inflammation, irregular menses and prostatitis etc. Compounds from the roots have exhibited excellent anticancer properties and Russo et al. [53] have demonstrated that natural extract from the roots (containing tannins-antioxidant properties) exhibits an inhibitory effect on all the cancer cells they examined.

### 3.2. Chlorophytum Borivilianum Sant. and Fern

One of the most common supplements used by dwellers in mountain ranges and deeper forests in many parts of India has been the herbaceous plant *Chlorophytum borivialinum* (Liliaceae; Figure 4c,d),the roots of which provide rich nourishment. The roots are eaten raw and possess aphrodisiac properties with no side effects. Now the extracts from these roots have yielded almost one hundred medicinal formulations. The major bioactive compounds are saponins and alkaloids and the drugs generated are used as effective treatments for the remediation of erectile dysfunction, spermatogenesis, diabetes and arthritis, among others. [54,55]. Pharmaceutically, *C. borivialinum* is very important because it is the source of 25 alkaloids, vitamins, minerals, proteins, carbohydrates, steroids, polysaccharides and albumin.

### 3.3. Coleus forskohlii (Willd) Briq

The leaves of this plant are consumed raw or cooked with other vegetables. These plants have been known to be medicinally important. The herb has been mainly used as an excellent medicine for treating heart and respiratory disorders in Ayurvedic medicine for centuries. Extracts are known to activate all hormone-sensitive adenylate cycle enzymes. Now we know that this herb is the only source of “Forskolin.” The tuberous roots of the plant produce labdane-diterpenoid forskolin, which is the main active compound. Minor diterpenoids, deacetylforskolin, 9-deoxyforskolin and a few more diterpenoids are also reported to be present in the roots of this species [56,57,58]. Antimicrobial, antioxidant and related pharmaceutically useful characteristics have been well tested internationally (in Egypt, Africa and in many European countries) within modern biotechnological parameters. While more than a hundred species of the genus *Coleus* (Family: Lamiaceae-mint family) are cultivated, the four species *C. amboinicus, C. forskohlii, C. spicatus* and *C. malabaricus* are found in nature.

Similarly, members of Anacardiaceae (Figure 1c,d) and Liliaceae provide seasonal food items with universal uses.

## 4. Food Ingredients

*Curcuma longa* (Turmeric): Turmeric is internationally famous, being the most ancient ingredient in Indian foods and food items including curries. Mostly famous for its antiseptic and resistance developing properties, the herb has been cultivated allover India for centuries and is also used for sacred worship. Much recent research has been validating ancient beliefs about this herb and now this “Indian Yellow gold” curcumin has been shown to be effective against many inflammatory and infectious diseases but its use as a drug has remained limited due to its low bioavailability and its rapid elimination from the body. Researchers have reported another new medicinal use for curcumin, the bioactive component of Indian kitchen spice turmeric (*Curcuma longa*). Nanoparticles of curcumin could offer a potential new treatment for tuberculosis (TB) that would be less prone to the development of drug resistance [59].

## 5. Precautionary Food Items

Local herbalists (*Vaidyas* in India) have suggested, and hence people in villages and remote forest areas have been using, the following plants in various forms (roots, leaves, whole plants in the form of juice, dried powder or paste)as precautionary food items. The list of such plants is quite exhaustive but just a few examples are essential. For example, *Albizzia lebbeck,* bark decoction is effectively used in leprosy and its leaves and seeds are used for eye troubles; *Bauhinia purpurea* is used for muscular pain, fever, headache and body swelling. Decoction prepared from the roots of *Bombax ceiba* was said to be effective in curing white discharge in urine. Some plants, commonly used for the treatment of joint inflammation are *Adhatoda vasica* (Basak), *Tinospora cordifolia* (Giloy), *Vitex negundo*. Medicines are prepared in the form of paste, powder, extract of plant parts and extracted oil. The list of such plants is quite exhaustive. In almost all parts of the world, the use of common native plant species for treating a variety of ailments has been a regular social practice. Some plant genera, viz., *Cinchona*, *Rauwolfia serpentina, Tamarindusindica, Zingiber officinale, Ocimum sanctum, Vitex negundo, Cassia tora, Amaranthus spinosusAndrographis paniculata, Swertia chirata, Amorphophallus paeniifolius, Calotropis gigantia, Cardiospermum helicacabum, Cymbopogon* sp, *Cissus quadrangularis, Vanda tessala, Alternantha sessilis, Cassia adnate, Sida cordata, Bauhinia purpurea, Argimone mexicana* are being used in local treatments for a variety of diseases and ailments. Local herbalists (*Vaidyas* in India) suggest using their roots, leaves andthe whole plants in the form of juice, dried powder or paste. Some plants commonly used for the treatment of joint inflammation are *Adhatoda vasica* (Adusa), *Tinospora cordifolia* (Giloy) and*Vitex negundo* (Nirgundi). Medicines are prepared in the form of paste, powder, extract of plant parts and extracted oil. Many plant species of these families are not regular food items but are known to serve as precautionary food additions in order to keep healthy. For earlier populations (a few thousand years ago), the leaves and roots in particular of the above plants must have served as staple food items. Many of these are still in use among tribal/isolated populations. For example, *Combretum* sp. and *Terminalia* sp. are used even now. The decoction extracted from the fruits and leaves of *Combretum trifollatum* Vent is used as reducing agent for intoxication from opium or similar drugs and is also taken as anthelmintic juice.

### 5.1. Drinks from Leaves

Since antiquity, the genus *Ocimum* (Figure 4b, known as Tulsi in India) has occupied a central place among herbs. The genus *Ocimum* by and large is not only known as sacred in India but also holds immense medical, metabolic and therapeutic potential as is described in the ancient Indian Ayurvedic text, Charaka Samhita. The designation of spp of *Ocimum* as the “Queen of herbs” was based on its wider use as a medicinal herb, with almost compulsory ritual of chewing a few leaves everyday. *Ocimum* varieties have been widely used in traditional Greek, Roman, Siddha and Unani systems of medicine. *Ocimum* is rich in phenylpropanoids, terpenoids and their derivatives, known for their therapeutic roles. The availability of the genome sequence now opens the possibility of identifying genes involved in producing therapeutic molecules and to produce them in vitro, as revealed by genomic data onfive different types of Tulsi (*Ocimum tenuflorium* subtype Rama, *O. tenuflorium* subtype Krishna, *O. gratissimum, O. saccharicum* and *O. kilimandscharicum).* The medicinal properties of *Ocimum* have been attributed to specialized compounds produced as a part of its defence mechanism. These compounds are called ‘metabolites’ because they are a by-product of the plant’s metabolism. There are more than 40 secondary metabolites in the *Ocimum* species, which are exploited by humans to treat diseases. Apigenin, taxol and urosolic acid are known to impart anti-cancer properties while citral is an anti-septic and eugenol has anti-infective properties [60,61]. Leaves of the *Mentha* species (Figure 4a) serve as a very strong pungent, effective part of a drink for promoting a good appetite. Often used in small quantities, the leaf juice is mixed with many vegetables and food items so as to be more digestive. Pharmaceutically (Table 2), this aromatic herb contains 60–70%carvone and about 10% limonene. Extracts possess antioxidant potential and protect against DNA damage. Total polyphenol extracted from 1 mg leaves equals 500 mcg of gallic acid [31].

*Stachytarpheta jamaicensis* is an annual herb that behaves also as a perennial plant, becoming bushy in several places [43], is eaten like spinach leaves and also consumed as a medicinal drink. This herb is also called Brazilian tea. Dried leaves are marketed for the treatment of many disorders like the removal of intestinal worms, venereal diseases, cardiac troubles etc. The leaves are prepared as a drink to protect against malaria and even lung infections.

### 5.2. Drinks from Roots

The roots of *Chlorophytum borivilianum* (Figure 4d), mentioned above, also serve as a good drink and in some parts of Nagaland people use it as a supplementary drink after the evening meal (personal experience). In African and Native American traditional medicines, the dried roots of *Podophyllum peltatum* and or *P. emodii* (also in the Indian subcontinent) were often used as drinks but now we have extracted podophyllin, which includes the active constituent podophyllotoxin. Similarly, species of the genera *Combretum* and *Terminalia* are known to be used, which have greater medicinal uses. There are a large number of plants, the roots of which serve as excellent food itemsand are also an excellent source of flavonoids, vitamins and proteins. The classical use of carrots (*Daucas)* and radishes *(Raphanussativus*) etc. is common knowledge.

## 6. Discussion

Dealing with the settlement phases of early human populations and related social cultural practices, this paper assumes that to begin with, migrating populations—through forests—must have found trees as the major choice for food because of flowers and fruits, and then shrubs and herbs would have served secondary needs. Obviously, migrating people must have acquired familiarity, and natural expertise, with selecting food items, particularly the plant parts (leaves/flowers/fruits/rhizomes and roots) of selected plant forms from among hundreds of wild trees/shrubs and herbs. They must have learnt this by losing many lives of their co-travelers on account of the poisonous effects of some, and the normal expected effects, of certain plant parts [36]. Time has been the greatest teacher of evolving and settling populations and for the regular availability of food-forms early civilizations practiced “cultivation,” evolving farming practices. For more than six thousand years, as per the records available in many countries including China, India and elsewhere [9,37,41], plants have offered valuable food items with both nutritional and medicinal value. Growing evidence from many modern laboratories supports the beneficial effects of plant bioactives (flavonoids etc.) that are naturally present in fruits and vegetables. These compounds exert effects on blood pressure and insulin control, suggesting that flavonoids might be more likely than other constituents of fruits and vegetables to explain the lower risk, particularly for cardiovascular disease and diabetes, which remain major contributors to morbidity and mortality. Several flavonoids may be important to health: specifically, anthocyanins that are present in red/blue colored fruits. Several compounds extracted from various parts of plants (leaves, small young twigs, roots and sometimes from the whole plant) such as several alkaloids—camptothecins, combretastatins, flavopiridol, podophyllotoxins, taxanes, vinca alkaloids, cell cycle target inhibitors ([13,14,15,16,17,21] see also Table 1 and Table 2)—are the main chemical constituents of some genera and species belonging to the Apocyanaceae, Meliaceae, Podophyllaceae, Combretaceae families and many more. Many microbiological trials on the extracts of a variety of these plants have been carried out [18,20,29,30,45,46,55,59,62,63,64,65,66,67] all over the world, which have finally been recommended for medicinal use. The modern pharmaceutical industry is becoming more than 60–70% based on thousands of such plants, which yield rich quality and quantity of compounds [15,18,37,46,62] and are exceedingly useful for manufacturing a large number of life-saving drugs.

### 6.1. Early Crops: Cultivation Practices

Several thousand years ago, most plants were wild. Our early civilizations started cultivating those useful plants, which were used as food crops (e.g., *Sorghum*) and also protected them and related which wild species had great medicinal use (e.g., *Sesamum*). As per available dispersed information, these were the earliest cultivated genera popularly known among cultivation practices prevalent since 4000 years BC. The genus *Sesamum* L is one of the oldest crops in the world now calledthe“queen of oilseeds.” The two species *Sesamum indicum* L and *S. mulayanum* Nair (Pedaliaceae) have been categorically designated as plants of high potential medicinal importance. Rich in oil (38–54%), proteins (18–25%), calcium, phosphorous and oxalic acid, the two species also possess lignans (sesamin, sesamolin and sesamol) compounds which are strong antioxidants [68,69]. Sesamin and sesamolin possess bactericide and insecticide properties can also inhibit the absorption and production of cholesterol in the liver. Trials on checking the growth of melanoma have also been encouraging, suggesting antitumor effects. *Sorghum* grains are quite rich in carotenoids and phenolic compounds. Cultivation of these and several other useful plants have been an inspirational source and within the past one hundred years we have observed enormous progress.

In modern approaches, we have combined multidisciplinary technologies and have extracted and identified specific chemical compounds for producing very particular medicines from plant parts. Practically, the modern pharmaceutical industry is based on 80 to 90% biological products (including animal parts discovered to be useful in medicines). Plants that yield appreciable quality and quantity of polysachharides, steroids, terpenoids, flavonoids, alkaloids and antibiotics are suitable for extracting compounds to be shaped as drugs for many ailments/disease, including cancer treatments. Medicinal plants are cultivated and cultured the world over. Nowadays, food is being improvised by mixing two or more crops or agricultural products. *Sorghum* flour is mixed with sweet potato [70] and the resulting product increases protein content and iron metabolism, thereby making an almost food-drug treatment for anemia. Phenolic compounds and carotenoids show high plasma total and antioxidant capacity.

### 6.2. Ethnomedicinal Information and Present Efforts

In almost all parts of the world tribes/isolates use native plants for treating common ailments—several of these, e.g., *Cinchona, Rauwolfia,* etc. (see, Table 1 and Table 2; Figure 1, Figure 2, Figure 3 and Figure 4) have been brought into the modern system of medicine. Almost all tribal populations in India have been using only local plants for treating common diseases. For example, the leaf powder of *Tamarindus indica* (ginger) and *Ocimum sanctum* (Tulsi) for coughs, *Vitex negundo* (Nirgumdi) for the common cold and *Cassia tora* for skin infections. The paste of *Rauwolfia* is sometimes used to cure insect, scorpion and snake bites. In gastric disorders, *Amaranthus spinosus* (cooked leaves) is commonly used. To treat malarial fever, they commonly use *Andrographis paniculata* (Kalmegh) powder with black pepper in equal amounts. For intermittent fever, *Swertia chirata* (Chiraita) is used. They have sound knowledge of medicinal plants and have identified several species that effectively treat rheumatoid arthritis. These include *Amorphophallus paeniifolius* (corms), *Calotropi sgigantia* (root bark), *Cardiospermum helicacabum* (Balloon vine) and *Cymbopogon* citrates (Lemon grass) etc. Several plants are used for setting bone fractures and in the orthopedic treatment of tribal herbal healers. The roots, stem and leaves of plants like *Cissus quadrangularis* (Harjora), *Vanda tessala, Alternantha sessilis* and theroots of *Cassia adnate, Sida cordata, Bauhinia purpurea* etc., are used for healing wounds for 10–15 days on broken bones. Isolated populations have conserved these plants in natural forests for orthopedic treatment. Dried powder in the form of the paste of *Argemone mexicana* is dried, powdered and made into a paste and then applied to infected portions of the skin and wounds. Plants like *Albizzia lebbeck,* bark decoction used in leprosy and leaves and seeds are used for eye troubles and *Bauhinia purpurea* is used for muscular pain, fever, headache and body swelling. Decoction prepared from the roots of *Bombax ceiba* to cure white discharge in the urine of tribal women is also conserved by ethnic people. Triterpenoid acids, such as oleanolic and ursolic acid, which are common plant constituents, are known to possess weak anti-inflammatory and anti-tumor activities. Attempts to synthesize new analogues with increased potencies have led to the synthesis of 2-cyano-3,12-dioxoolean-1,9-dien-28-oic acid (CDDO) and its methyl ester, which have potent in vitro and in vivo anti-tumor activity against a wide range of tumors including breast carcinomas, leukemias, and pancreatic carcinomas. The World Health Organization (W.H.O.) has recognized about 20,000 medicinal therapeutic plants in the world of which [35,71] includes a greater percentage of those plants which we have been using as food items for centuries. Conservation of germplasm and cultivation of all edible plants(because of their recently discovered pharmaceutical properties) [22,23,24,25,26,27,28,72,73,74] (see tables and Figure 1, Figure 2, Figure 3 and Figure 4) will not only generate income for farmers but also improve the health care system of the entire human race.

### 6.3. Excess of Everything is Unhealthy!

There is a saying, “one’s meal is another’s poison.” This statement evolved after the very long wandering experience of moving populations several centuries ago. Plants have antioxidant systems for self-protection against biotic and abiotic stress conditions. As is well established, the most important function of antioxidants is reducing reactive oxygen species (ROS). ROS are a byproduct of respiration. They can be beneficial to the human body by removing pathogens and old proteins. However, an overproduction of ROS can cause various factors such as environmental pollutions, stresses, synthetic substances etc. [46]. They could eventually be responsible for chronic diseases including aging, cancer, cardiovascular diseases, rheumatoid arthritis, atherosclerosis, etc. Certain nutrients ingested in large quantities, more so vitamins acting as drugs but some combinations, for example vitamin A derivative acutane causes abortions. Excess consumption of vitamins A and C induces many defects [72] in the developing foetus hence pregnant women are often advised for only prescribed doses. On the other hand, malnutrition and vitamin deficient diet further results in abortions. The truth is even larger that, one liter (1000mL) of milk is almost more than a half diet, excess of milk results in many intestinal and digestive problems. Additionally, the inherited genetic allergies to proteins, carbohydrates, lipids and several essential aminoacids (e.g., Phenylalanine; glutamic acid etc.) are very much prevalent in all human populations thus depriving them from many food items.

Needless to say, the list of chemical compounds extracted by modern pharmaceutical research from these food-plants confirm that ancient food habits were health oriented. Modern pharmaceutical researches have extracted a large number of chemically active organic compounds which are often approved after rigorous microbiological tests [12,24,25,30,37,41,45,52,61,63,71,72,73,74,75] for preparing medicines. Obviously, these drugs or new generation of chemical compounds weaved in the form of medicines, extracted from the food items cannot be consumed as food (in bulk every day). Obviously, “Food can be Medicine but medicine cannot be used as Food.”

## 7. Summary

“Food” is the primary vital need of each and every living organism. Food is “the substance digested by an individual to suppress the basic vital instinct of hunger.” The hunger demands inflow of those compounds which on ingestion serve as the energy-fountain supplying required vitamins, sugars, proteins, minerals and adequate quantity of water, for performing vital physiological functions. The early human populations had had first depended on plants and plant parts to satisfy their hunger; and some of them simultaneously must have had additionally discovered small animals to meet their requirement. The so called medicinal plants, as we know today, are those plants which constitute our major part of food and majority of them are loaded with highly nutritive compounds. This healthy food concept then evolved, has been maintained and transferred over hundreds of generations. By now, it is well established that chemicals extracted from plants have a wide range of pharmacological applications; possess tendency to produce many kinds of secondary metabolites. These metabolites are polyphenols, flavonoids, terpenoids, steroids, quinones, alkaloids, polysaccharides and so on. Most of these compounds have properties which prevent and cure various diseases as well as aging in mammals including humans.

Modern technological advancements assisted with biochemical, microbiological and genetic—toxicological tests have totally revolutionized present day pharmaceutical industry and life saving drugs from several plants, which are often used as a part of our diet, have been generated. Thus on the basis of uses the food has been grouped into three categories: *1. Main food:* which satisfies hunger; *2. Supplementary food:* the type or form of food which, is used as additional or subsidiary to the main food (such as fruits, salad and healthy drinks-milk, juice etc.)); *3. Food items*: This category should include (i) Precautionary food; (ii) Food ingredients.

## Figures and Tables

**Table 1:** Edible fruits on the trees in the wild and chemical compounds extracted from plant-parts.

Name of the Plant	Common Uses	Chemical Compounds
***Aegle marmelos*** (L.) Corr. Family: Rutaceae English Name: (Figure 1a)	Pulp used as an additional dietary requirement in Summer; fruit-pulp eaten raw. Dysentery Sunstroke-Juice extracted from the fresh leaves—taken orally; Excellent supplement with food and vegetable; used as best medicine to cure	Aeglemarmelosine, marmelosin, alloimperatorin, marmelide, tannic acid, marmin, umbelliferone, isoimperatorin, isopimpinellin, skimmin, marmesin, marmesinin, fatty acids, beta-sitosterol
***Annona reticulata*** L. Family: Annonaceae English Name: Bullocks heart (Figure 1d)	Creamy pulp is nutritious, cooling and quenches thirst in fever.	Anonaine, 7-1actone acetogenin, *cis-/trans-isomurisolenin,* along with six known cytotoxic acetogenins, annoreticuin, annoreticuin-9-one, *cis-/trans-bullatacinone,* bullatacin, *cis-/trans-murisolinone,* and squamocin from ethyl acetate extract of seeds [22]
***Annona squamosa*** L. Family: Annonaceae English Name: Custard Apple (Figure 1c)	The fruit possesses astringent, cooling, anti-scorbutic and febrifugal	The diterpenoidalkaloidatisine is the most abundant alkaloid in the root. Other constituents of *Annona squamosa* include the alkaloids oxophoebine, reticuline, isocorydine, and methylcorydaldine and the flavonoidquercetin-3-O-glucoside [23]
***Buchnania lanzan*** Spreng. Family: Anacardiaceae English Name: Buchanan’s Mango (Figure 2b)	The fruits are useful in treating leprosy, skin diseases, burning sensation, cardiac debility, abdominal disorder and constipation.	Flavonoid, alkaloid
***Cordia dichotoma*** G. Forster. Family: Boraginaceae English Name: Sestan Plum	It is astringent, anthelmintic, diuretic, demulcent and expectorant.	Allantoin, beta -sitosterol and 3’,5-dihydroxy-4’-methoxy flavanone-7-O-alpha-L-rhamnopyranoside.
***Diospyros melanoxylon*** Roxb. Family: Ebanaceae English Name: Coramandel Ebony Persimmon (Figure 1b)	The fruit is carminative and astringent. The leaves are diuretic, carminative, laxative and styptic.	Flavones, Triterpenes [24]
***Carissa congesta*** Wight Family: Apocynaceae Local Name: Karonda English Name: Christ’s thorn	The fruits have a pleasant taste and are slightly laxative	Carissone, β-D-glucoside. Quercetin, kaempferol, leucoanthocyanins and vanillic acid.
***Feronia limonia*** (L.) Swingle Family: Rutaceae English Name: Wood apple (Figure 1e)	Cures cough, dysentery, heart diseases, vomiting; blood impurities, good for throat, asthma, opthalmia, leucorrhoea.	Methyl Chavicol, Thymol, Limonene and Linalool
***Ginkgo biloba*** L. *(a gymnosperm-huge tree)* (Figure 3a,b)	Ovules are eaten in Korea, Japan and China since antiquity	Excellent taste, digestive and protein source Ginkogolides, Diterpene
***Grewia asiatica*** L. Family: Tiliaceae	The fruit is astringent, cooling and stomachic.	Carbohydrates, proteins, fatty acids and other active metabolites like flavonoids, tannins, phenols, alkaloids, steroids and triterpenoids, lignans, lactones, flavones, anthocyanins etc. [25,26]
***Madhuca latifolia*** J.F. Gmelin. Syn. *M.indica* J F Gmel. Family: Sapotaceae English Name: Indian butter tree (Figure 2a)	This small very nutritious fruit is the basic food for most of the tribes in Central India for centuries. Leaves are astringent, flowers are used in cough, bronchitis and kidney complaints.	Sugar, vitamin, protein, alkaloids, phenolic compounds, Vitamins A & C. Good source of α- and β-amyrin acetates. Arachidic, linolelic, oleic, myrisic, palmitic and stearic acids, α-alanine, aspartic acid, cystine, glycine, isoleucine and leucine, lysine [12]
***Moringa oleifera*** Lamk. Family: Moringaceae English Name: Drum stick tree	Leaves are eaten as supplement to main food; often preferred for use in malnutrition, anaemia, asthma, anti-inflamatory, anthelmintic and ophthalmic	Alkaloids, Flavonoids, Anthocyanins, proanthocyanidins and cinnamates.
***Manilkara hexandra*** (Roxb.) Dubard Family: Sapotaceae English Name: Obtuse leaved Mimusops (Figure 2d)	The stem bark is boiled and used as tonic and astringent. antiinflammatory, antiulcer, antidiabetic, antibacterial	Starch, terpenoids, proteins, anthraquinoneglycoside, cardiac glycoside, saponins and tannins in bark [27]
***Pithecellobium dulce*** (Roxb.) Benth. Family: Mimosaceae English Name: Madras thorn, Manilla tamarind	Analgesic and anti-inflammatory (Sahu and Mahato, 1994).	Saponins and flavanoids
***Phoenix sylvestris*** Roxb. Family: Arecaceae English Name: Wild Date Palm (Figure 2c)	Fruits are edible. Mostly prevalent in dry land; most favourite fruits for children	Vitamin B complex, Ascorbic acid; Fruits contain fibers as well.
***Phyllanthus emblica*** L. Family: Euphorbiaceae English Name: Indian gooseberry	Fruits consumed raw as well as cooked; often prepared prickles and tonic pastes have been the commonest supplementary to main food	Excellent source of Vitamin C, Ascorbic acid Asthma, bronchitis, bleeding gums, scurvy, diabetes, anaemias, infection, weakness of memory, stress, tension and loss of hair.
***Syzygium cumini*** (L.) Skeels Family: Myrtaceae Local Name: Jamun English Name: Black berry	Fruits are eaten raw; very tasty and known since antiquity as very effective tonic and medicine.	Fruits are used as an effective medicine against diabetes, heart and liver trouble. Seeds posses anti-inflammatory, anti-arthrtic, antipyretic, anti-bacterial, anti-fungal and analgesic activity [28] Contain Iron, Oleanolic acid and triterpenoids
***Tamarindus indica*** L. Family: Caesalpiniaceae Local Name: Imali English Name: Tamarind (Figure 1f)	Asthma, amenorrhoea, ulcers, fever, helminthiasis, wounds, jaundice, ringworms and laxative.	Calcium, iron, vitamin B,C, and phosphorous. Pectin, tartaric acid and dihydroxybutanedioic acid
***Terminalia bellirica*** (Gaertn) Roxb. Family: Combretaceae Local Name: Baheda English Name: Baleric myrobalan	Indigestion, diarrhoea, piles, leprosy, dropsy, biliousness, headache and fever.	Triterpenoids, Flavonoids, triterpines, sterols, phenolics and Lignans, [29]
***Terminalia chebula*** Retz. Family: Combretaceae Local Name: Harra English Name: Chebulic myrobalan	Not as part of food but consumed as a tonic to get relief from jaundice, colic, cough, asthma, haemorrhoids, diarrhea, spleen and liver disorder [30]	Chebulinic acid, tannic acid, gallic acid, resin, anthraquinone and sennoside

**Table 2:** Common wild plants (herbs) used as precautionary medicinal food—items by isolated population/tribes in India.

Name of the Plant	Routine Usage	Chemical Composition
***Achyranthes aspera* L.** Family: Amaranthaceae English Name: Prickly-chaff flower	Leaves are cooked as vegetable ;Pungent, astringent, diuretic, pneumonia, dysentery, Fever, Asthma and poisonous bite	Alkaloid achyranthine and betaine.
***Argemone mexicana* L.** Family: Papaveraceae English Name: Prickly poppy	Not consumed as food but very effective in certain ailements as prescribed by *local vaidyas*	Dropsy, jaundice, cutaneous infection, diarrhoea, dysentery, leprosy, cough and wounds.
***Centella asiatica* (L.) Urban** Family: Apiaceae English Name: Indian penny wort	Leaves are boiled or made paste for eating as a tonic.	Asiaticosides, Asiatic and madecassic acid Effective in curing Anxiety, neurosis, memory enhancer, jaundice, leprosy, rheumatism, bronchitis, asthma and kidney trouble
***Coccinia indica* Wight & Arn.** Family: Cucurbitaceae English Name: Ivy Gourd (Figure 2f)	Skin diseases, bronchitis, diabetes	Resin, alkaloids, starch, glucose, gum, fatty acids, carbonic acid, calcium, iron and phosphorus.
***Curcuma longa* L.** Family: Zingiberaceae English Name:Turmeric	Used for centuries as a major additive powder for preparing vegetables and curry items; used as antiseptic	Curcumin, demethoxycurcumin, bisdemethoxycurcumin and Curcuminoids
***Daucus carota* L.** Family: Apiaceae English Name: Carrot	Regularly eaten as a vegetable; modern salad always includes carrot Appetizer, carminative, digestive, diuretic, anthelmintic, cardiotonic	Beta-carotene, α-terpinolene, α-pinene, β-pinene, myrcene and myristicin. Appetizer, carminative, digestive, diuretic, anthelmintic, cardiotonic ophthalmic, anorexia and bleeding gums
***Gentiana kurroo* Royle** Family: Gentianaceae English Name: Indian Gentian	Casual food supplement to Improve appetite, stomachic, fever and blood purifier	Gentiopicroside, xanthones, monoterpene alkaloid; polyphenol and flavones
***Hibiscus sabdariffa* L.** Family: Malvaceae English Name: Red sorrel	Mixed with vegetable Diuretic, choretic, hypotensive, debility, conjunctivitis, swelling and inflammation.	Diuretic, choretic, hypotensive, debility, conjunctivitis, swelling and inflammation-contain-Anthocyane, flavonoids and mucilages
***Ocimum sanctum* L.** Family: Lamiaceae English Name: Holy Basil (Figure 4b)	Leaves almost regularly used by many persons since ancient times. Serves as precautionary food item among traditional medicines for Cough, bronchitis, catarrh, wounds, anorexia, ophthalmopathy, anthelmintic, skin and genitor-urinary disorder.	Terpenes and Eugenol
***Mentha spicata* L.** Family: Lamiaceae English Name: Spearmint (Figure 4a)	Leaves always mixed with simple food items of breakfast; Leaves also made paste to mix with vegetables and curries. Known to cure constipation, diarrhea and nausea, headaches, migraines, nervous strain, fatigue and stress, asthma, bronchitis, catarrh and sinusitis.	This aromatic herb contains 60–70% carvone and about 10% limonene. Extracts possess antioxidant potential and protect DNA damage. Total polyphenol extracted from 1 mg leaves equals 500 microgram of gallic acid [31] Medicinal compounds: a-pinene, b-pinene, carvone, 1,8-cineole, linalool, limonene, myrcene, caryophyllene and menthol.
***Momordica dioica* Roxb.ex Willd** Family: Cucurbitaceae English Name: Spine gourd	Astringent, diuretic, laxative, antiasthmatic, antipyretic, antidiabetic, aphrodisiac, antihemorroidal, hepatoprotective, bleeding piles and urinary complaints	Alkaloid, steroids, tritepenoids
***Phyllanthus amarus* Schumach. & Thonn.** Family: Euphorbiaceae English Name: Carry Me Seed, Black catnip, Child pick-a-back	Jaundice, stomachic, diuretic, diarrhoea	Phyllanthin and hypophyllanthin
***Paspalum scrobiculatum* L.** Family: Poaceae English Name: Kodo Millet	In dry seasons in almost all villages and extreme rural areas in Northern India grains are used as an alternative to rice Kodo millet is a nutritious grain.	Protein, fibre, carbohydrates, calcium, and also contain high amounts of polyphenols, an antioxidant compound
***Panicum miliaceum* L.** Family: Poaceae English Name: Proso Millet, Hog Millet or White Millet.	It is sold as health food, and due to its lack of gluten, it can be included in the diets of people who cannot tolerate wheat.	Protein, calcium, lysine, arginine, glycine and alanine.
***Solanum nigrum* L.** Family:Solanaceae English Name: Black Night-shade	Leaves are eaten as vegetables in poor areas of tribes in Jharkhand and Bastar (Indian states).Leaves and small fruits are taken as raw but only when advised.	Psoriasis, ringworm, rheumatism, gout, dysentery, colitis and skin disease Alkaloids solanine, solamargine, saponin
***Zinziber officinale* Roscoe** Family: Zingiberaceae English Name: Ginger	Used as an additive food item to be mixed with vegetables curry and drinks for centuries	Gingerol Excellen remedy with honey to cure cough and cold
***Termitomyces eurrhizus* (Berk.) Heim.** **Syn. *Termitomyces albuminos* (Berk.) Heim.** Family: Tricholomataceae English Name: Termite mushroom (Figure 3f)	Edible mushroom found wild in the forest floor where enough humus retains after rainy season. Due to high proteinaceous nutrient content many edible mushrooms are cultivated world over.	Basically rich in aminoacids, particularly lysine; higher protein and fibers content with vitamin D; a substance lentinan (a beta glucan); inhibits tumour ontogeny; rich in phosphorous and potassium; too lees caloric value makes mushrooms even more favoured dish in modern food items.
